# Ion Translocation Driven by Electric Field Generated in Excited-State Reactions

**DOI:** 10.1021/acs.accounts.5c00434

**Published:** 2025-08-14

**Authors:** Hao-Ting Qu, Alexander P. Demchenko, Igor O. Koshevoy, Pi-Tai Chou

**Affiliations:** † Department of Chemistry, 33561 National Taiwan University, Taipei 10617, Taiwan, Republic of China; ‡ Institute of Physical, Technical and Computer Sciences, Yuriy Fedkovych National University, Chernivtsi 58002, Ukraine; § Department of Chemistry, University of Eastern Finland, Yliopistokatu 7, 80101 Joensuu, Finland

## Abstract

The fundamental mechanism of ion translocation against the concentration gradient in biological systems has become a central focus of research. The variation of the electric field in response to external stimuli can be an essential trigger in this process. The introduction of molecular machines has enriched this field by providing a direct approach to converting energy into mechanical work. However, existing models mainly rely on photoisomerization dynamics that alter the location of ion-carrying molecular segments to achieve transportation. In a recent series of works, we present a new design of light-driven anion-translocating molecular machines that do not involve any conformational changes. In the designed structures, the dramatic redistribution of positive charge from the electron acceptor to the donor moiety in the dipolar cation dye is driven by excited-state intramolecular charge transfer (ESICT). This shifts the anion binding site to the opposite side of the molecule, facilitating a fast and directional ion motion. The continuous reversible cycle arises from the fact that the forward motion occurs during the excited-state lifetime on the high-energy potential energy surface, whereas the reverse reaction proceeds on the ground-state potential energy surface. Thus, the light quanta not only provide the energy source but also serve as the factor that drives the ion in the specified direction.

The unexpected observation about the anomalous dual-emission behavior of various phosphonium and pyridinium salts in nonpolar solvents has prompted the proposal of such a photoinduced counterion migration mechanism. Unlike the ultrafast ESICT process, which occurs on a subpicosecond time scale, the appearance of a strongly Stokes-shifted emission band—attributed to anion translocation—is observed over tens to hundreds of picoseconds. Furthermore, it was shown that the increase in ion radius results in the retardation of anion motion, which can be adequately explained by the mechanism we proposed. The interpretation of ion motion as a relaxation process toward electrostatic equilibrium is supported by the observed monoexponential decay of the spectral response function *C*(*t*) that is commonly used to describe the dynamics of solvent relaxations. Based on *C*(*t*) analysis, the dependence of the motion rate on the temperature and solvent viscosity demonstrated the absence of significant energy barriers during the process. Through structural modification of functional groups, the appended photoinduced intramolecular proton-transfer group anchored on the donor side enhances the efficiency of ion translocation.

In this Account, we briefly summarize recent reports on photoinduced counterion migration and highlight its potential for enabling transmembrane ion transport. Although challenges in future practical applications still need to be addressed, the core principle of modulating the directionality of anion migration along the dipolar cationic backbone via ESICT offers a promising opportunity for a concise and general design strategy for molecular machines that simulate the active translocation of ions in biological systems.

## Graphical abstract

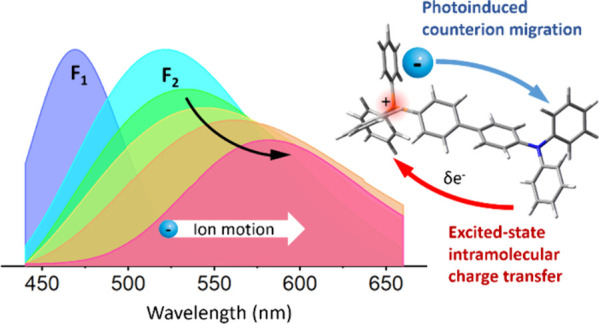

## Key References

-

Belyaev, A. ; Cheng, Y. H. ; Liu, Z. Y. ; Karttunen, A. J. ; Chou, P. T. ; Koshevoy, I. O. A Facile Molecular Machine: Optically Triggered Counterion Migration by Charge Transfer of Linear Donor-π-Acceptor Phosphonium Fluorophores. Angew. Chem., Int. Ed. 2019, 58 (38), 13456–13465.10.1002/anie.20190692931291049 ^1^ Examines the anomalous emission spectra of phosphonium salts in nonpolar solvents and proposes the mechanism of counterion migration to justify the observations.
-

Lin, T.-C. ; Liu, Z.-Y. ; Liu, S.-H. ; Koshevoy, I. O. ; Chou, P.-T. Counterion migration driven by light-induced intramolecular charge transfer. JACS Au 2021, 1 (3), 282–293.34467293 10.1021/jacsau.0c00107PMC8395631 ^2^ Pyridinium salts also support our hypothesis about the counterion migration in 2019.
-

Belyaev, A. ; Su, B. K. ; Cheng, Y. H. ; Liu, Z. Y. ; Khan, N. M. ; Karttunen, A. J. ; Chou, P. T. ; Koshevoy, I. O. Multiple Emission of Phosphonium Fluorophores Harnessed by the Pathways of Photoinduced Counterion Migration. Angewandte Chemie 2022, 134 (19), e202115690.10.1002/anie.202115690PMC930677935146862 ^3^ Investigation of the effect of the additional migration channel on the photophysical properties.
-

Chang, K. H. ; Yang, Y. H. ; Su, K. H. ; Chen, Y. ; Lin, T. C. ; Li, J. L. ; Liu, Z. Y. ; Shi, J. H. ; Wang, T. F. ; Chang, Y. T. ; Demchenko, A. P. ; Yang, H. C. ; Chou, P. T. Light Induced Proton Coupled Charge Transfer Triggers Counterion Directional Translocation. Angew. Chem., Int. Ed. 2024, e202403317.10.1002/anie.20240331738578721 ^4^ Introduction of the proton-transfer switch into the system can effectively accelerate the counterion migration rate.
-

Qu, H.-T. ; Partanen, I. ; Chang, K.-H. ; Lin, Y.-D. ; Koshevoy, I. O. ; Belyaev, A. ; Chou, P.-T. Insights into the photoinduced anion translocation of donor−π–acceptor^+^ (ion)^−^ molecules. Chemical Science 2024, 15 (47), 20045–20055.39568931 10.1039/d4sc04738aPMC11575608 ^5^ Quantification of the counterion migration rate and elimination of the possible occurrence of other intrinsic reactions during counterion migration through further kinetic analysis.

## 1 Introduction

Living cells possess the ability to provide the motions of ionic species across their membranes in the required direction with great specificity and under stringent regulation by different external effectors. These processes being of great functional importance are performed by specific proteins demonstrating a great variety of structures.^6^ Their ion translocating functions vary from providing a passive mode of mobility to the active transport occurring in out-of-equilibrium conditions and against the electrochemical gradients.^7,8^ To circumvent the principle of microscopic reversibility, which states that any chemical reaction should occur in both forward and backward directions along the same mechanistic pathway, such functions of ion transporters require the input of energy. The energy is supplied in a coupled chemical reaction (such as ATP hydrolysis^9^) but can also be provided by absorption of light quanta (as in rhodopsins^10^). The process of ion motion is of extreme complexity, involving the cascades of coupled binding-release steps with different groups of atoms. Molecular structures of many ion transporters are known. However, even with the knowledge of all their details on the molecular level with atomic resolution, it is hard to understand the mechanisms of these reactions in terms of their driving force, directionality, and rates.^9^ Without such understanding, modeling of the complex behavior or even of the basic steps of these processes becomes a major problem.^11,12^ In this respect, the molecular machine concept offered the stimulating direction of research activities. The principles were established on how to make autonomous molecular motions unidirectional in a cyclic way and to perform their cycles continuously. Operating with a minimal number of synthetic components, it was shown that external energy input in the form of light quanta may enable the thermodynamically unfavorable processes such as the repeated and directional motion of molecular cargo.^11,13,14^ In order to function autonomously and continuously, the forward and return motions should follow different routes, always forming a cycle. Essentially, for accomplishing the cycle, the energy of the light quanta may not be directly involved in driving the motion. The role of reactions of excited-state isomerization was in changing the energy profile along the reaction pathways and in leading reversibly to less-stable isomers.

Synthetic molecular configurations were suggested for the powered translocation of ions.^15–17^ Using isomerization dynamics of photochromic units inserted into molecular structures allowed the ion binding, transportation, and release operation to be achieved in bilayer membranes. However, the shuttling motions that were realized demonstrated only passive transmembrane transport. The excitation energy was largely dissipated in space, resulting in quasi-equilibrium diffusional mobility.

In view of the fact that electrostatic interactions modulate the properties of ion channels^18,19^ and ion-transporting ATPases^20^ and that they may present the most essential driving force for ion motion, the attempts to model these effects using synthetic systems of minimal size appeared quite reasonable. An extended dye molecule should have an electrostatic ion-binding site that can be occupied in the dark. Upon the absorption of a light quantum by the dye electronic system, excited-state intramolecular electronic charge transfer (ESICT) occurs, resulting in a change in its dipole vector, which can be designed in a way that creates, extends, or even reverses it. The electrostatic environment of the bound ion is thus altered accordingly. The former binding site disappears, and the new high-affinity site appears at a distance, creating the electrostatic driving force for moving the ion to a new binding site.

The circular machine motion can be composed of two sections: the forward excited-state process and the reverse ground-state process in the dark (Figure 1). The whole circle is powered by a single light quantum. Such a system may demonstrate two types of the cycles, one with counterion bound to its initial binding site and the other with it traveling to a new location. Due to the strong influence of ion position on the ESICT energy, these motions may be detected by the change in fluorescence spectra. In our work,^1–5^ this concept was applied to a series of ionic phosphonium and pyridinium chromophores, where due to ESICT, the electronic charge was transferred to the other side of the molecule, driving directional counterion motion via electrostatic interactions.

**Figure 1:**
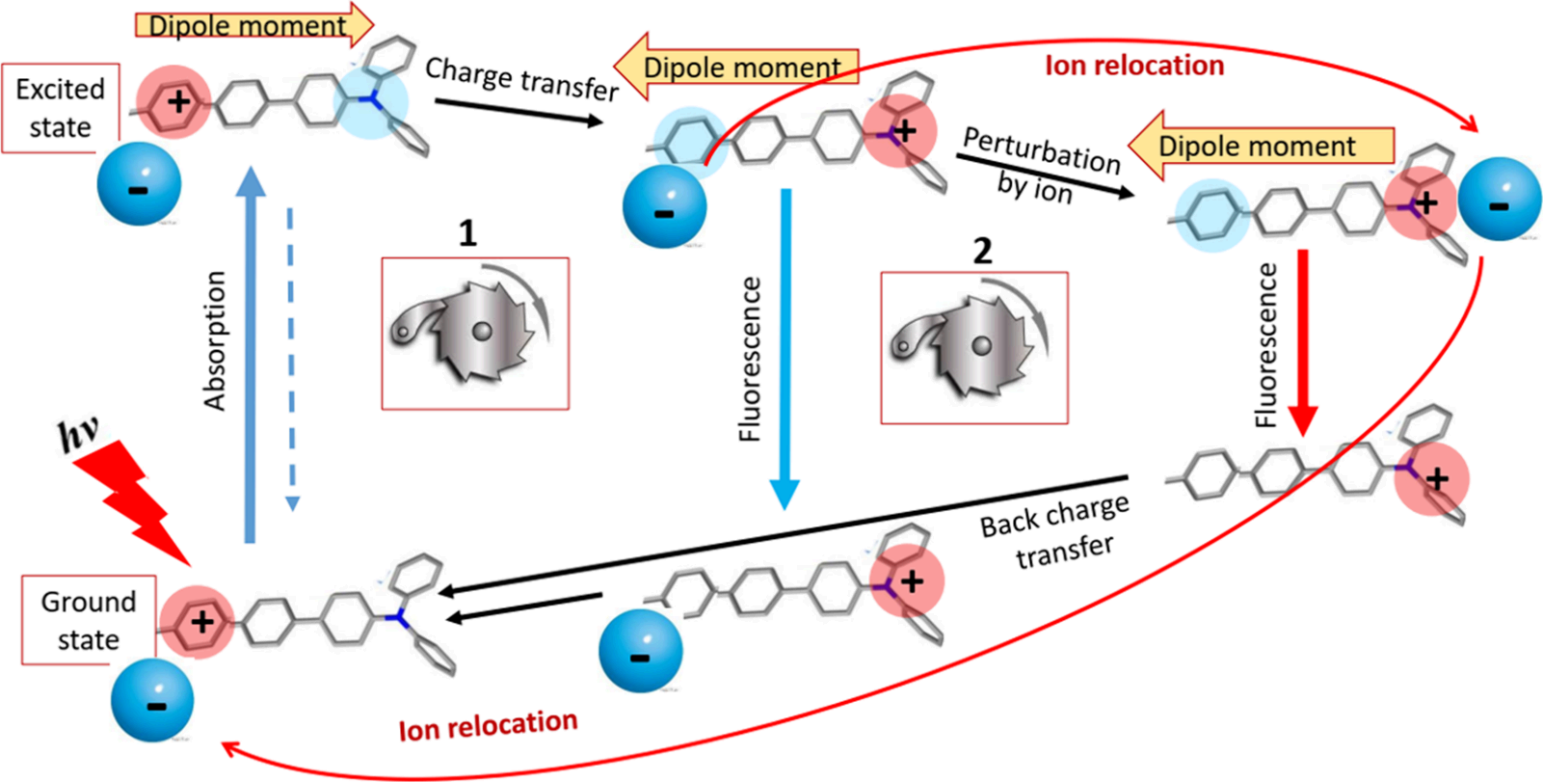
Realized scheme of the electronic charge transfer reaction driving the ion motion. The absorption of light quantum results in redistribution of charge, generating the large dipole moment. Fluorescence emission and the following back transfer returns fluorophore to its initial state. The process is unidirectional due to the strong energy gap between ground and excited states. Redistribution of electronic charge abolishes the primary ion-binding site but creates a new site due to the appearance of a positive charge at the opposite side of molecule, creating the conditions for directed anion motion. Two types of cycles can be realized. (1) The ESICT results in strongly Stokes-shifted fluorescence emission, and its ultrafast rate does not allow the ion to move. (2) Ion translocation occurs during the excited-state lifetime. Its binding to a new site formed by ESICT results in strong perturbation of the electronic state, leading to a new fluorescence band further shifted to the red.

This Account addresses the analysis of this novel concept in molecular machine action, which is based on relocation of its electrostatic binding site during the excited-state reaction and on its application for photoinduced directional ion migration. We illustrate the great power of steady-state and time-resolved fluorescence spectroscopy to resolve the energetic and kinetic aspects of ion motion. On this basis, it becomes possible to analyze the effects of distance between the binding sites, of the ion size, and of external factors such as solvent viscosity and temperature on this motion. Furthermore, this effect can be enhanced by combining ESICT with excited-state intramolecular proton transfer (ESIPT), opening an additional dimension of proton-coupled charge transfer for photoinduced ion migration.

## 2 Observation of Photoinduced Ion Translocation

In 2019, we conducted the studies of solvatochromism on a series of push–pull phosphonium salts,^1^ which consisted of a phosphonium electronic acceptor connected to an arylamine electronic donor by varying the phenylene π-spacers (Figure 2a–c). Their emission spectra displayed a typical characteristic of dipolar charge transfer (CT) dyes: bathochromic shift of fluorescence spectra with increasing solvent polarity. Unexpectedly, in the cases of low-polarity solvents such as toluene, an anomalous dual emission was observed. Both emission bands demonstrated significant Stokes shifts associated with ESICT, but their simultaneous presence was quite unusual and required further analysis. It was derived that the extension of the phenylene spacer between the donor (D) and acceptor (A) not only increases the D/A distance but also reduces the Coulombic interaction between anion and positively charged donor moiety, thus prolonging the time constant of ion translocation. For convenience, we assigned the higher-energy band as F_1_ and the lower-energy one as F_2_. The time-resolved results showed a continuous spectral transformation from the F_1_ to F_2_ band, rather than conversion between two distinct emission bands (Figure 2d,e), as it happens with molecular relaxations in polar liquids. Such transformation was retarded with the increase of counterion size (Figure 2f) so that the slower motion of larger ions was observed by a decrease of the spectral evolution time constant (decay time constant monitored at F_1_).

**Figure 2:**
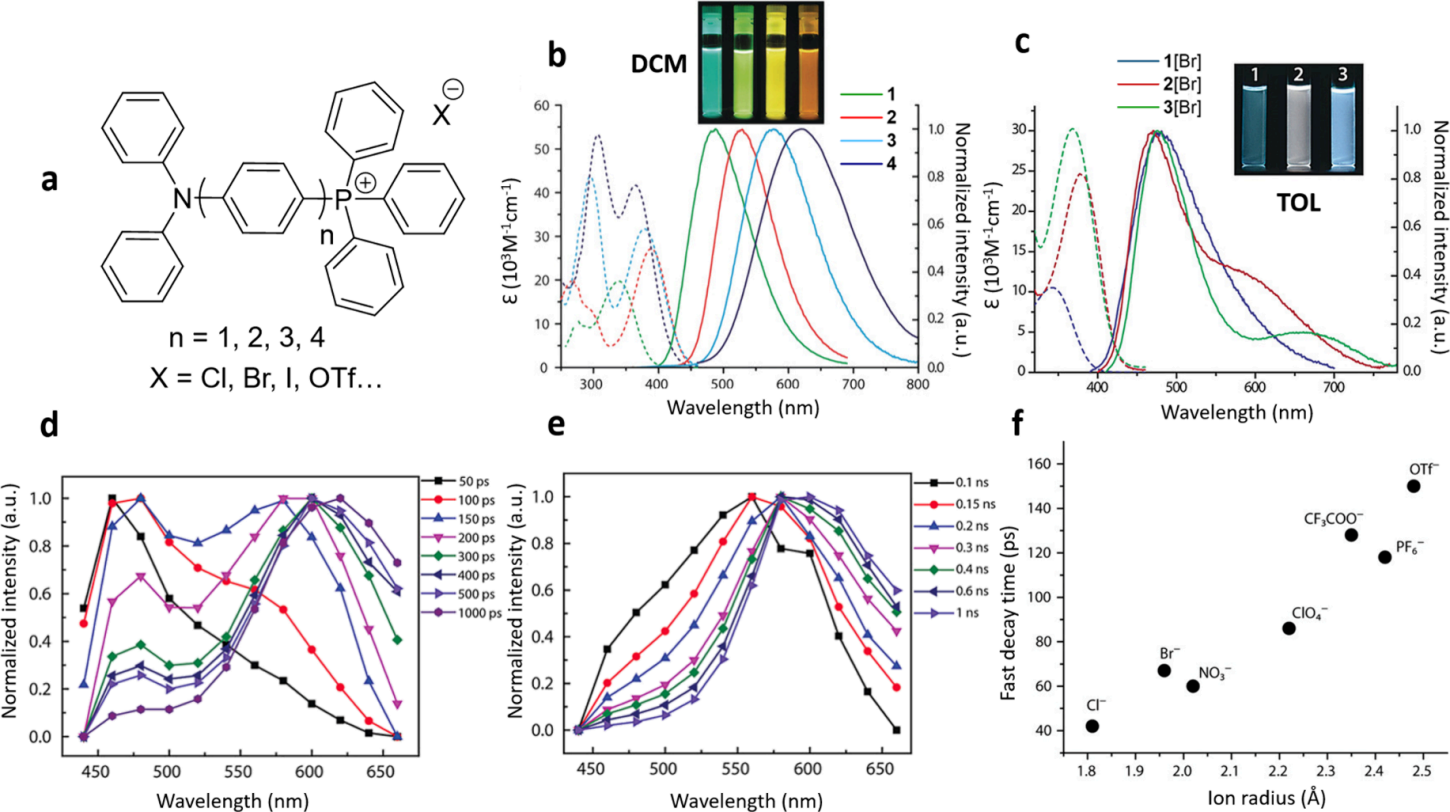
Monitoring of anion translocation by steady-state and time-resolved fluorescence spectra.^1^ (a) The structures of phosphonium salts ***n*[X]** with different spacer lengths. (b) The steady-state absorption (dashed) and emission (solid) spectra of the phosphonium salts in dichloromethane (DCM), indicating the stronger Stokes shifts with a spacer increase. The photo shows the corresponding solutions under UV light. (c) The steady-state absorption (dashed) and emission (solid) spectra of the phosphonium bromide salts in toluene (TOL) demonstrating the appearance of the F_2_ band for the dyes with shorter spacer lengths. (d, e) The time-resolved emission spectra of **2­[Br]** (d) and **2­[OTf]** (e) in toluene at 298 K. (f) The fast decay time constant monitored at the F_1_ band for **2­[X]** in toluene as a function of radius of the anions. Reproduced with permission from ref 1. Copyright 2019 John Wiley and Sons.

In subsequent studies, the research was extended to a series of dipolar pyridinium cationic chromophores to reinforce the understanding of the counterion translocation via the combination of experimental and computational approaches.^2^ Through detailed fluorescence lifetime measurements, we ruled out the possibility that the conformational changes in the cationic chromophore, such as the twisted intramolecular charge transfer (TICT),^21^ could be the origin of dual emission. Additional evidence based on modifications of cation structures was obtained in support of the assertion that the counterion migration gets involved in the dynamics of fluorescence spectra. Only a single band was observed for the compounds, in which the ion binding did not occur due to the absence of charge transfer^1^ or due to the structural restrictions.^2^ Besides, the previous report^1^ showing that the rate of ion motion decreased with an increase in the anion size and the length of the π-bridge was reconfirmed. We thus obtain a simple system that allows observation and control of the directional translocation of anions driven by absorbed photons and occurring during the lifetime of excited states.

## 3 Structural Modifications Generating Multipolar ESICT

To gain in-depth insight, we utilized the unique connectivity of the phosphorus atom that affords creating multipolar molecules with a variable number of arms carrying the electron donor groups.^3^ This allowed the achievement of a transition from a dipolar to multipolar configuration in the excited state and thus provides additional pathways for anion motion. The variation of phenylene π-spacers increases the range of such possibilities. Our synthesized compounds were categorized based on the number of branches and the spacer lengths of each branch. Cations possessing identical arms were named symmetrical cations, whereas those with differing arms were unsymmetrical cations. The notation of these samples uses numbers to represent the lengths of spacers and subscript numbers, indicating the number of arms. The anions **[X]** were used, where **[X]** ^**–**^ = **[Br]** ^**–**^ or **[OTf]** ^**–**^. The principle of modulation of anion motion and the results for some compounds of these series are presented in Figure 3a.

**Figure 3:**
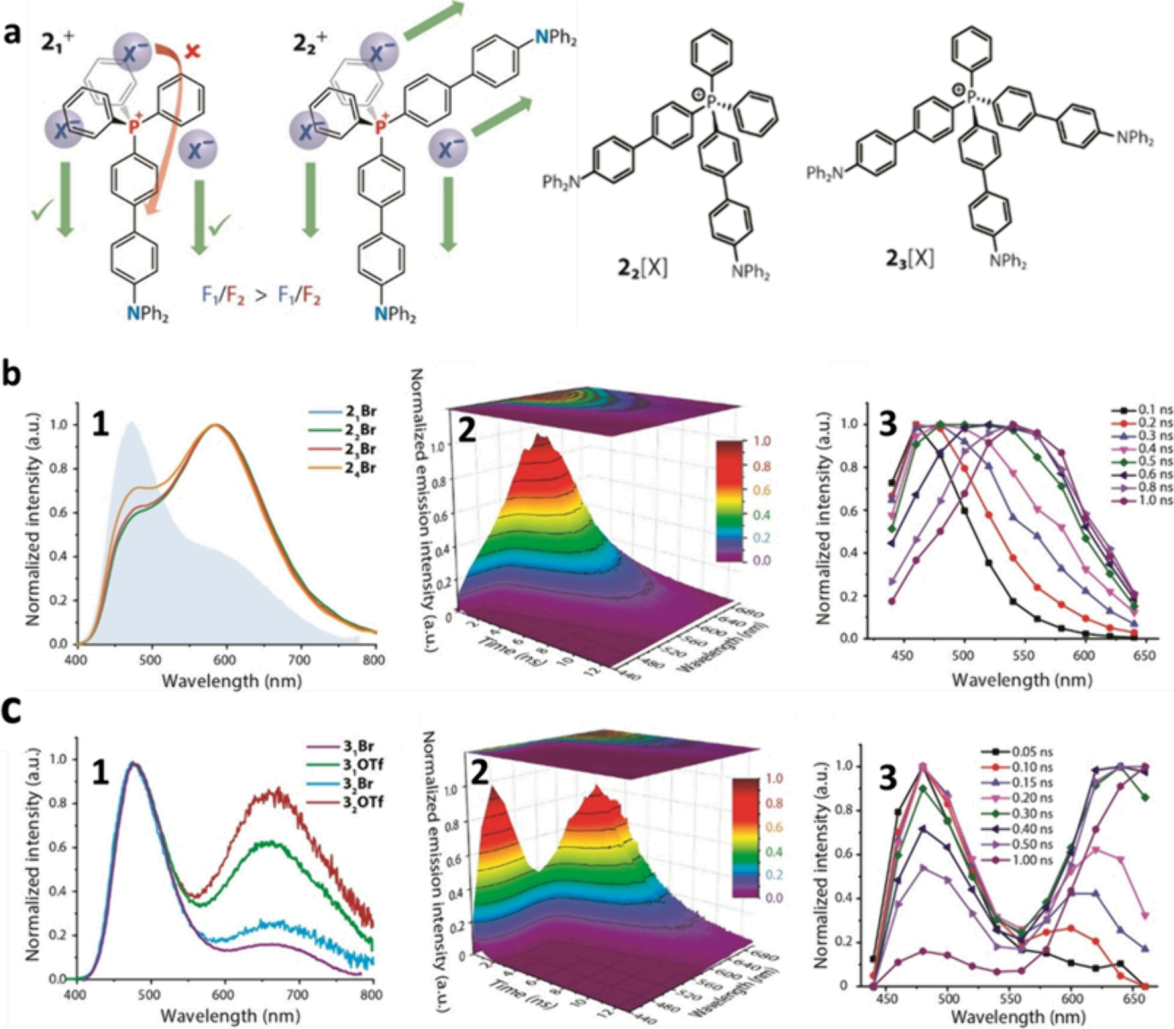
Branched ESICT dyes generating transportation of anions.^3^ (a) Schematic representation of the effect of dipolar and quadrupolar architectures on the possibility of anion migration and the structures of typical dyes. (b) Normalized emission spectra of **2**_***n***_ **[Br]** (1). Filled graph for its symmetrical analogue **2**_**1**_ **[Br]** is presented for comparison. Time-resolved emission spectra of **2**_**2**_ **[OTf]** in toluene at 298 K (2, 3). (c) Normalized emission spectra of **3**_***n***_ **[X]** (***n*** = 1, 2; **[X]** ^**–**^ = **[Br]** ^**–**^ or **[OTf]** ^**–**^) (1). Time-resolved emission spectra of **3**_**2**_ **[OTf]** in toluene at 298 K (2, 3). Reproduced with permission from ref 3. Copyright 2022 John Wiley and Sons.

In polar solvents, upon optical excitation, the initially delocalized multipolar charge distribution across the entire chromophore undergoes reorganization, transforming into a localized dipolar charge transfer state concentrated on one of the branching arms. We observed that, in the polar solvent DCM where ions dissociate, the charge-transfer emission is associated with the lowest dipolar excited state, which is in line with Kasha’s rule and corresponds to the arm with the longest spacer. Therefore, the steady-state emission spectra feature a single emission band, similar to single-armed reference compounds. The symmetrical multipolar cationic chromophores, such as **2**_***n***_ **[Br]**, manifest the phenomenon of excited-state symmetry breaking.^22–24^

The situation is different in weakly polar solvents, where the multipolar character of the phosphonium cation in the excited state has a distinct influence on the photoluminescence behavior, reflecting the location and dynamics of ions. In the steady-state spectra, the new symmetrical **2**_***n***_ **[X]** dyes demonstrate high-energy (F_1_) and low-energy (F_2_) emission bands of variable relative intensities (Figure 3b). Compared with the correspondent dipolar dye, the quadrupolar **2**_**2**_^**+**^ architecture in the presence of **[Br]** ^**–**^ anion increases the intensity of the F_2_ band (λ = 586 nm) dramatically relative to the F_1_ band (λ = 468 nm). Probably, by providing two accessible migration pathways, the symmetrically branched molecular structure of **2**_**2**_^**+**^ enhances the probability of photoinduced anion translocation. The kinetics of continuous transformation of spectra extends over the whole picosecond range, indicating the directional ion motions. As expected, in frozen solutions, no ion motions occur and therefore we observe only F_1_ band in emission.

When the dyes differing in number and length of substituents are compared, it becomes possible to extend the spectrum over the whole wavelength range producing the white-light emission to achieve analysis for the strong separation between F_1_ and F_2_ bands. The results for representative dyes demonstrating a highly efficient band separation are presented in Figure 3c. In this and similar cases, the relative intensity of the F_2_ band increases substantially in comparison with their dipolar analogs. The time-resolved spectra, providing the measure of the rate of ion translocations, demonstrate a very strong redistribution of excited-state energy on these events.

## 4 The Enhancing Effect by Proton Transfer Dynamics

The charge distribution of the cation governs not only the direction of counterion migration but also its migration rate. The anion binding strength at the electron donor terminal of our molecular machines can be enhanced by introducing a site that provides coupling between the excited-state intramolecular proton transfer (ESIPT) and the ESICT reaction.^4^ In the suggested configuration, the deprotonated hydroxyl electron donor group generated by ESIPT counterbalances the original positive charge at the acceptor site, subsequently concentrating the positive charge on the protonated morpholine group. Here, the role of proton transfer is different from that presented in the literature, where the intermolecular photoinduced proton release acts as a trigger mechanism for molecular machines and switches.^25^ In contrast, in ESIPT, the proton donor and acceptor moieties reside in the same molecule and are hydrogen-bonded to each other, so that the proton transfer coupled with an intramolecular electronic rearrangement may not result in the formation of charged species or irreversible chemical transformations.^26^ Instead, in this configuration, the driving force for producing work on stepwise ion translocation can be enhanced by the proton-coupled charge transfer (PCCT) reaction, generating the more informative gigantic shifts of fluorescence spectra.^4^

The results of the PCCT work^4^ are illustrated in Figure 4. A linear pyridinium-expanded biphenyl salt 1-hexyl-4-(4′-hydroxy-3′-(morpholinomethyl)-[1,1′-biphenyl]-4-yl) pyridin-1-ium perchlorate, **BPym–OH/ClO**_**4**_, was synthesized. It shows a strong intramolecular hydrogen bond between −OH and the morpholine nitrogen site that allows the ESICT-ESIPT coupled reaction, leading to transformation to a tautomer structure with a strong charge transfer character. In a weakly polar solvent, such as toluene, the formed tautomer generates anion migration to gain further stabilization. Initially, with a rise-time constant of ∼200 fs, the consequent fluorescence displayed a strongly red-shifted band maximum F_T_ at ∼640 nm (cf. absorption maximum at 360 nm), resulting from the proton-transfer tautomer with a large charge transfer character.^26^ Meanwhile, a new emission due to anion translocation appeared with a substantial delay as an ultrabroad, red-shifted band F_T‑ion_ with the maximum at ∼750 nm and extending further to the near-IR region.

**Figure 4:**
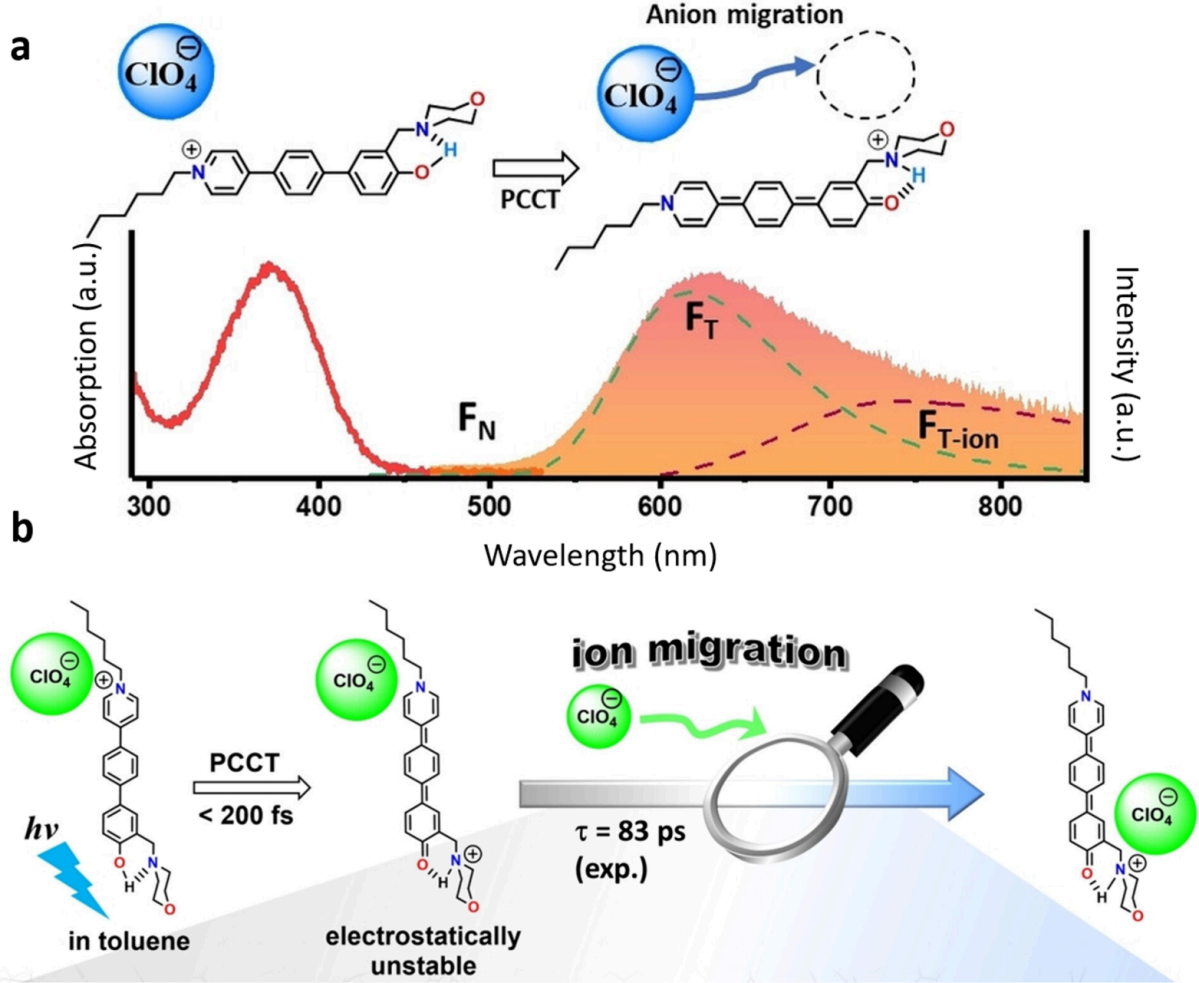
Anion translocation driven by the proton coupled charge transfer (PCCT) reaction in the cationic organic dye and its spectroscopic manifestation.^4^ (a) The smart dye 1-hexyl-4-(4′-hydroxy-3′-(morpholinomethyl)-[1,1′-biphenyl]-4-yl) pyridin-1-ium perchlorate, **BPym–OH/ClO**_**4**_, studied in toluene absorbs light at 370 nm with negligible initial emission F_N_ performing an ultrafast PCCT reaction (*k*_PCCT_ ∼ (200 fs)^−1^). As a result, the emission becomes intense and strongly red-shifted with a band F_T_ at 640 nm. Counterion binding to a new site results in the appearance of a very broad F_T‑ion_ band with a maximum at 750 nm with a much slower rate *k*_T‑ion_ ∼ (83 ps)^−1^. (b) The sequence of events occurring on ion translocation after PCCT. Reproduced with permission from ref 4. Copyright 2024 John Wiley and Sons.

This emission band was attributed to the migration of the ClO_4_^–^ ion to a new location, driven by reorganization of the electric field upon the ESICT-ESIPT coupled reaction, which shifts the more positive charge to the morpholinomethyl site. In comparison, the control derivative that does not relocate the anion shows only F_T_ emission, and the one that cannot perform the ESIPT reaction displays both the emission F_N_ and the ion-stabilized ESICT emission F_N‑ion_ at much shorter wavelengths. Time-resolved analysis and advanced molecular dynamics (MD) simulation results support the fact that the driving force for ion relocation generated by ESIPT-enhanced excited-state electronic charge redistribution is much stronger than that of the ESICT reaction itself.^4^ The new 750 nm band rises at ∼83 ps, manifesting the time constant of the anion translocation by 1–2 orders of magnitude slower than that of the typical solvent relaxation occurring in common liquid solvents (a few picoseconds).

Proton coupled charge transfer plays an important role in biological systems,^27^ where many membrane-active proton-anion cotransporters^28^ and proton-cation antiporters^29^ have been discovered, but the coupling mechanisms of these charge transfer events are still unclear. With our current level of knowledge, we may suggest that the electronic polarization component must be important here, opening new areas for research, analysis, and speculation.

## 5 Counterion Motion and Molecular Relaxations

Directed ion motion in solutions occurs against the background of stochastic processes of molecular diffusion,^30^ which must be better understood in order to convert these motions into useful work. Operating with phosphonium and pyridinium salts in diverse solvents, we tried to establish the basic mechanism of the relaxation process leading to the stabilization of excited-state energy as a result of the appearance of anions at the sites created by ESICT.^4,5^ It is known that the electronic polarization of the medium in response to a change of electric field produced by the charges and dipoles is an universal factor, whereas the relaxation of surrounding dipoles by their rotational and translational motions is characteristic of polar environments.^31,32^ In polar low-viscous liquids, the dipole-orientational relaxations are terminated within early picoseconds.^33^ Here, we observe the migration process extending across the whole picosecond time range, occurring by 2–3 orders of magnitude slower than the solvent relaxation. Under this condition, the ESICT reaction typically proceeds on an ultrafast time scale of <1 ps, allowing us to bypass the its coupling with ion motion. In contrast, such reactions occurring in polar liquid environments are coupled with solvent relaxations and proceed much slower.^34^

The excited-state relaxations in media, such as the ionic liquid solutions^35,36^ and pure ionic liquid,^37,38^ have been represented by the spectral response function. In these systems, the relaxations generated by ions were also much slower than the common solvent response. The result of our studies^5^ suggest that a single ion can be responsible for the relaxation leading to dramatic reduction of the excited-state energy and correspondent shifts of fluorescence spectra. We explain its origin by the effect of electrochromism (intrinsic Stark effect) that is known for ion-dipole interactions in solutions^39,40^ to influence the chromophore ground and excited electronic state energies.^41^ This fact of inducing electrochromism by rapidly moving a single ion was previously not described in the literature and therefore deserves special consideration.

Additional arguments for the proposed mechanism of ion motion were derived from detailed time-resolved studies. Based on the decay/rise time constant at a specific wavelength,^1–3^ the strong dependence on counterion size, spacer length, and donor–acceptor combination of structures was observed. In order to thoroughly elucidate the complex dynamics of ion motion, we applied the more advanced theoretical models^42^ based on spectral response function *C*(*t*)^5^ describing the shifts of spectra as a function of time:^43^

$$C\left(t\right)=\frac{\nu \left(t\right)-\nu \left(\infty \right)}{\nu \left(0\right)-\nu \left(\infty \right)}$$ 1

where ν­(*t*) denotes the time-dependent emission peak position in cm^–1^. Here, ν(0) and ν(∞) are the initial and equilibrium peak positions, respectively. In terms of energetics, *C*(*t*) designates the irreversible processes of evolution in time of the solvation free energy toward equilibrium. In our study, we deconvolved the time-resolved spectra of **3­[OTf]** (Figure 5a,b) with two Gaussian functions, designating one as the high-energy band, which basically remains stationary over time, and the other as the low-energy band, which undergoes a red shift.^5^ The spectral response function was derived from the latter, generally exhibiting single-exponential behavior with a time constant on a scale of hundreds of picoseconds. (Figure 5c)

**Figure 5:**
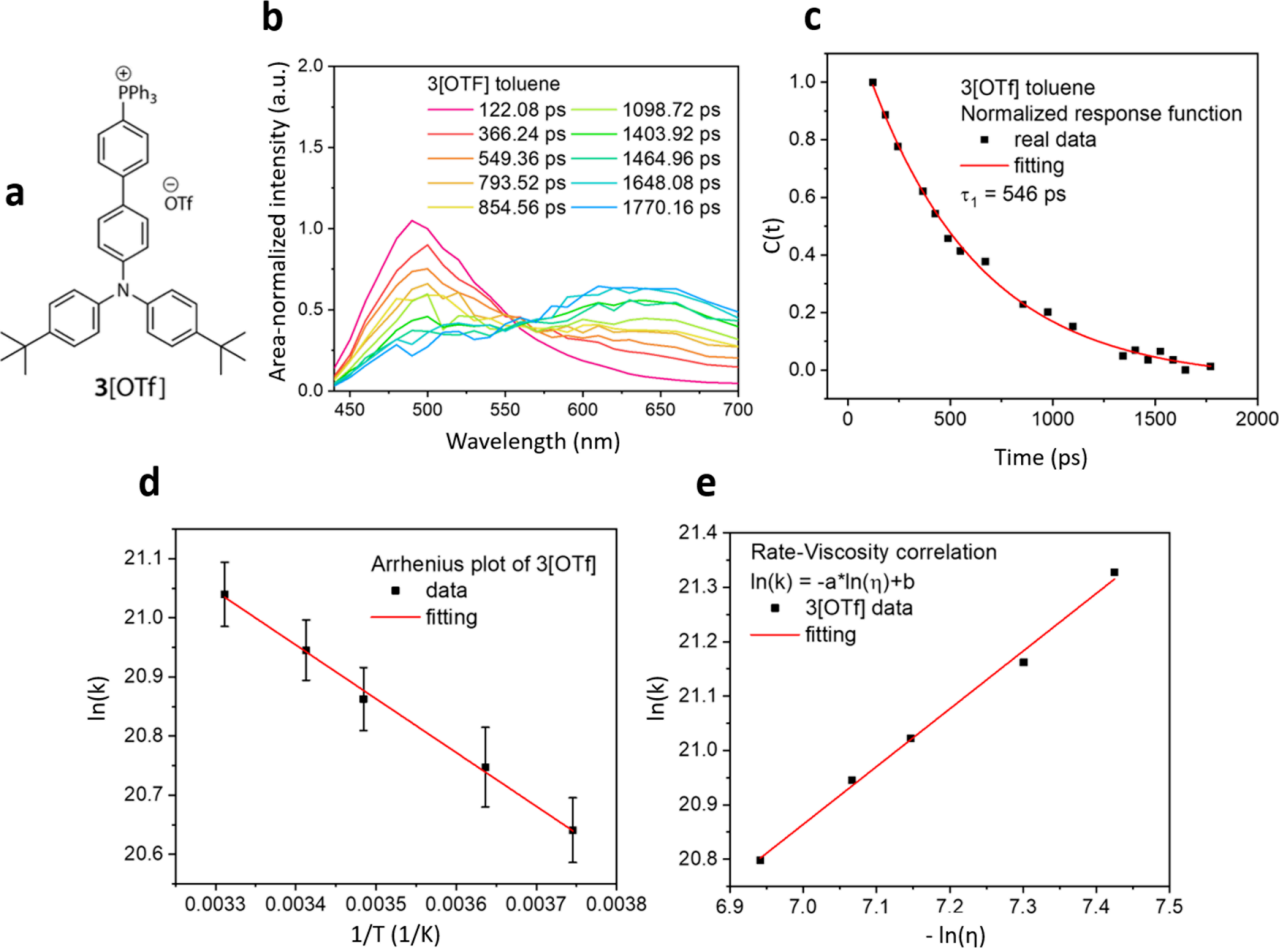
Analysis of dynamics of ion translocation.^5^ (a) The structure of **3­[OTf]**. (b) Time-resolved emission spectrum of **3­[OTf]** in toluene. The spectrum are plotted under the condition that the area of the entire spectrum is normalized to a constant value.^46^ (c) The spectral response function *C*(*t*) of **3­[OTf]** in toluene. (d) The plot of ln­(*k*) as a function of 1/*T*. (e) The plot of ln­(*k*) as a function of −ln­(η). *k* is the migration rate of **3­[OTf]**; *T* is the temperature on the Kevin scale, and η is the viscosity. Reproduced with permission from ref 5. Copyright 2024 Royal Society of Chemistry.

Using *C*(*t*) as a tool, we performed the temperature-dependent experiments in the iso-viscosity media (Figure 5d) and achieved the Arrhenius plot showing a basically barrierless reaction profile and a frequency factor of ∼10^10^ Hz, which is smaller than the common cases with intramolecular vibration frequency. At room temperature, we observed that the relaxation process is impeded by the viscosity of solvents (Figure 5e), exhibiting a similar dependence on viscosity as described by Kramers’ theory.^44,45^ This suggests that the friction generated by fluctuating solvent molecules serves as the main factor determining the relaxation rate.

## 6 Conclusion and Perspectives

Our experiments have clearly shown that the energy of light quanta can serve not only as the trigger but also as an essential driving force for machine action determining the direction and length for translocation of ions. By operating with a series of designed cationic dyes exhibiting the ESICT reaction, we demonstrated the discovery that the relocation of the anion binding site in the excited state induces an electrostatic force, consequently driving the anion motion. The absorption of single light quantum is sufficient for performing a continuous four-step cycle (Figure 1) that involves the excited-state and the ground-state energy surfaces, and the great difference in their energies excludes the possibility for reverse reactions. Operating only with electronic properties, this mechanism of molecular machine action does not require any large structural changes. Therefore, the electronic transformations and the driven motions of ions are fast, occupying the range of picoseconds. Its major advantage is the ability to directly observe the ion translocations in the energy and time domains using steady-state and time-resolved fluorescence spectroscopy and in this way determine its dependence on many external factors.

The proposed artificial molecular machines accomplish a reversible working cycle with the most minimal molecular design. Compared to rotaxane, where the macrocycle is mechanically interlocked on the thread, the above elaborated systems constrain the motion of ions only through Coulomb interaction within the ion pair, reducing the structural complexity. However, the ingeniously provided modifications may lead to substantial improvements. One of them was realized by appending the ESIPT site,^4^ which allowed an increase in the strength of the ion binding site formed in the excited state together with enhancement of the fluorescence response. Regarding the enlargement of the π-electronic system, the possibilities are limited, since according to the energy gap law,^47^ the shift of spectra to the red will be accompanied by a decrease of fluorescence yield mainly due to the increase of vibronic coupling and hence the internal conversion rates.

From this perspective, our molecular machines provide a potential strategy for the uphill ion transport delivering a repetitive work output, as the ion translocation is directly manipulated by the charge distribution of cationic chromophore but not by the concentration gradient of ions. For this, the bound ions should not exchange with the bulk environment. In our case, only low-polar solvents can minimize the ion presence in the solvent and eliminate the formation of solvation shells, thereby reducing the screening effect on the interactions between ions and their binding sites. Other possibilities, such as structure confinement of ions in heterocyclic structures, can be realized.^48^ It is worth noting that the ion dehydration is important for its transduction by natural^49^ and synthetic^50,51^ ion channels and pumps.^8^ Addressing the physical modeling of basic events puts forward the manipulation of artificial ion pumps and their environments. This can aid our understanding of the transport processes in biological systems.

Progressive ion-translocating work can be realized only in the cases when the ions will not return to their initial position in a reaction cycle but move to external sites of higher affinity. The step during which the interaction imposed on the anion decreases with the decay of excitation energy leaves the anion without electrostatic confinement in a low-polar environment. Therefore, it provides sufficient driving force to migrate to external binding if such bindings are formed in heterogeneous systems such as micelles, lipid bilayers, polar surfaces, etc. This may result in the operation of artificial systems forming the transmembrane gradients of ion concentrations with energy-storing capability. Synthetic molecular machines powered by light are still remote models of the processes occurring in biological systems. Therefore, we believe that the proposed highly general “minimalist” model, which reveals the importance of Coulomb interactions in ion migration driven by electronic conversion, deserves further development, analysis, and exploration.
